# Novel antitumor mechanism-of-action of an IL-2 fusion protein mediated by tumor associated macrophage repolarization and innate-like CD8+ memory T cells

**DOI:** 10.1186/2051-1426-2-S3-P123

**Published:** 2014-11-06

**Authors:** Wenxin Xu, Jack O Egan, Bai Liu, Xiaoyue Chen, Lijing You, Kaiping Han, Warren D Marcus, Lin Kong, Monica Jones, Emily K Jeng, Peter R Rhode, Hing C Wong

**Affiliations:** 1 Altor BioScience Corp., Miramar, FL, USA

## 

ALT-801, a fusion of interleukin-2 and a single-chain T cell receptor domain specific to a p53 peptide/HLA-A*0201 complex, has shown immune cell-mediated activity in human xenograft tumor SCID mouse models and in patients with metastatic malignancies. To further investigate the antitumor efficacy of this fusion protein in immunocompetent mice, we conducted mechanism-of-action studies in an orthotopic murine muscle invasive bladder cancer model. ALT-801 treatment (1.6 mg/kg i.v., day 7, 10, 14, 17 post-tumor instillation) significantly prolonged survival of C57BL/6 mice bearing orthotopic MB49luc bladder tumors when compared to equivalent treatment with either IL-2 (0.4 mg/kg) or PBS (8 mice/group; median survival: ALT-801, 48.5 days vs. IL-12, 36 days; vs.PBS, 33.5 days, p < 0.001), consistent with previous findings in xenograft tumor models. Tumor-bearing mice that were cured by ALT-801 treatment were also protected from subsequent MB49luc tumor re-challenge 2-3 months later indicating induction of memory immune responses. Studies in this model using immune cell depletion and KO mice revealed that ALT-801-mediated antitumor activity was dependent on CD4^+ ^and CD8^+ ^T cells and macrophage but not NK cells and required expression of IFN-γ (based on survival endpoint: PBS vs. ALT-801 or ALT-801+anti-NK1.1 Ab depletion, p < 0.05; PBS vs. ALT-801+CD8 Ab, CD4 Ab or clophosome depletion, P > 0.05; PBS vs. ALT-801 treatment of tumor-bearing IFN-γ or IFN-γR KO mice, P > 0.05). Consistent with these results, IHC, adoptive cell transfer and expression array studies showed that ALT-801 treatment led to activation and proliferation of CD4^+ ^and CD8^+ ^T cells which migrate from the lymphoid tissues to the tumor site where they secrete IFN-γ. Twenty hours after ALT-801 administration, tumor associated-macrophages (TAM) in the bladder of tumor-bearing mice were also transiently repolarized from an M2 (tumor promoting) to an M1 (tumor killing) phenotype in an IFN-γ dependent manner. Additionally, in vivo ALT-801 treatment induced innate-like CD8^+^CD44^high ^memory T cells to proliferate and upregulate NKG2D receptors. These cells may contribute to elevated splenocyte cytotoxic activity against mouse and human bladder tumor cells that was seen after ALT-801 administration. The results of these studies suggest that ALT-801 induces IFN-γ-dependent TAM repolarization and non-specific CD8^+ ^memory effector T cells that promote robust and rapid antitumor activity in mice bearing orthotopic MB49luc bladder tumors. This novel immunostimulatory mechanism-of-action appears to be distinct from that of IL-2 or other T cell-based immunotherapeutics and is currently being assessed in bladder cancer patients under treatment with ALT-801.

